# Editorial: Functional foods for metabolic health

**DOI:** 10.3389/fnut.2026.1821416

**Published:** 2026-03-30

**Authors:** Omar Guzmán-Quevedo, Jailane de Souza Aquino

**Affiliations:** 1 Laboratory of Experimental Neuronutrition and Food Engineering, Tecnológico Nacional de México (TECNM)/Instituto Tecnológico Superior de Tacámbaro, Tacámbaro, Michoacán, Mexico; 2 Centro de Investigación Biomédica de Michoacán, Instituto Mexicano del Seguro Social, Morelia, Michoacán, Mexico; 3 Postgraduate Program in Neuropsychiatry and Behavioral Sciences, Federal University of Pernambuco, Recife, PE, Brazil; 4 Laboratory of Experimental Nutrition, Department of Nutrition, Health Sciences Centre, Federal University of Paraíba, João Pessoa, PB, Brazil

**Keywords:** functional foods, inflammation, metabolic syndrome, obesity, phytonutrients, prebiotics, probiotics

Alterations in metabolic homeostasis are responsible for the development and high prevalence of a wide range of medical conditions, which have significant economic and social impact (1, 2). The development of obesity, hypertension, dyslipidemia, and glucose intolerance, which can result in cardiovascular complications and type 2 diabetes (T2D), poses a major challenge for healthcare systems worldwide (2). Metabolic disorders are primarily caused by the consumption of high-calorie diets rich in saturated fats and refined sugars (3). These diets, which are prevalent in modern food culture, are deficient in prebiotics (such as polyphenols and fiber) and probiotics (such as *Lactobacillus* and *Bifidobacterium*), which promote metabolic health (3, 4). Functional foods containing probiotics, prebiotics, and post-biotic agents have come to be a promising alternative to pharmacological therapies for these health problems (4). Functional foods have been shown to promote metabolic health (4), but there is still a significant gap to overcome in establishing effective therapies based on functional-based nutritional approaches.

The purpose of this Research Topic was to gather clinical and preclinical studies investigating the benefits of functional foods or ingredients in metabolic diseases. Thirty-three articles from studies examining functional effects on obesity, liver dysfunction, diabetes, cardiovascular diseases, hyperuricemia, and related conditions were included in this Research Topic.

The most investigated aspect was the relationship between obesity and probiotic and prebiotic consumption. This relationship was directly addressed by eight articles. First, Jia F. et al. examined the impact of *Flammulina velutipes* mycorrhizae dietary fiber on mice that were exposed to high-fat diet (HFD). The treatment prevented the obesity-related change by neutralizing the hepatic lipid metabolism change caused by HFD. In another study, Liu et al. evaluated whether dietary uridine could improve hepatic lipid metabolism in mice exposed to a HFD. The use of uridine resulted in a decrease in body weight (BW) gain, adiposity, and blood and liver triglyceride content. The treatment had a positive impact on the altered expression of genes involved in lipid transport and low-density lipid receptor (Ldlr). According to these findings, uridine could be used to treat obesity by reducing hepatic changes in energy metabolism. De Matteis et al. studied the specific contribution of extra-virgin olive oil (EVOO) to the anti-obesity effects of the Mediterranean diet. The authors found that EVOO is independently associated with lower body mass index (BMI) and waist circumference. The authors propose that consuming EVOO at least 6 days per week, with a daily consumption of 25 g, regardless of diet, can help prevent visceral adiposity.

Fermented foods are associated with metabolic benefits. Pihelgas et al. investigated how eating fresh and fermented vegetables (FV) can affect gut microbiota and body composition in individuals with normal BMI. By analyzing the microbiota profile before and after FV consumption, the study discovered that FV promoted a healthy gut microbiota composition, with an increase in butyrate-producing and anti-inflammatory bacterial species. According to this data, consuming FV, which is a source of fiber and lactic acid bacteria, is a promising strategy for promoting gut and metabolic health. In line with this, 2 weeks of FV consumption decreased the fat mass percentage compared to baseline measurements. A paper produced by Zhu et al. tested the functional effects of a radish seed glucosinolate extract in HFD-induced obese mice. BW gain was prevented by the treatment and serum lipids and lipid deposition in the liver were reduced. The gut microbiota diversity increased, leading to a decrease in *Faecalibaculum* and an increase in *Allobaculum, Romboutsia, Turicibacter*, and *Akkermansia*. Furthermore, the decrease in serum levels of ALT and AST suggests that there is no toxicity caused by the treatment.

As previous studies have demonstrated a reduced level of thiamine (vitamin B1) in obesity, a study tested its functional effects in mice exposed to a high-fat and high-fructose diet. Thiamine reduced BW gain and improved glycolipid metabolism in a dose-dependent manner. The positive outcomes were associated with dysbiosis protection, which was demonstrated by the rise in beneficial *Bifidobacterium pseudolongum* abundance and a reduction in potentially pathogenic *Proteobacteria* and *Ruminococcus gnavus* in the study (Xia et al.). Using a rat model of obesity, Thang et al. evaluated the anti-obesity effects of the probiotic bacterium *Limosilactobacillus reuteri* strain HM108 isolated from breast milk. Rats exhibited a dose-dependent beneficial response in several metabolic parameters including reduced BW gain, blood lipid content, and inflammation status. The treatment also showed a reduction of the *Firmicutes*/*Bacteroidetes* ratio. Thus, the use of *Limosilactobacillus reuteri* HM108 represents an interesting alternative to combating obesity. Flavonoids are recognized as modulators of energy metabolism impacting BW. Guzmán-Quevedo et al. reviewed literature that demonstrates how flavonoids and gut microbiota work together to regulate energy balance and prevent obesity development. Flavonoids, a type of polyphenols, can regulate the composition of the microbiota; it promotes a beneficial microbiota profile and increasing the production of short-chain fatty acids by bacteria. Favorable microbiota can metabolize flavonoids in metabolites. Then, both short-chain fatty acids and flavonoid-derived metabolites modulate energy metabolism in metabolically active tissues, including the liver, skeletal muscle, adipose tissue, and hypothalamus.

Four papers investigated in a general manner the functional effects of prebiotic components on metabolic disorders. Gu et al. developed a review showing the role of alteration in the ubiquitin-proteasome system (UPS) in the pathogenesis of lipid accumulation, inflammation, oxidative stress, and insulin resistance, which are involved in the progression of metabolic disorders. This review highlights UPS as a target for developing anti-metabolic disorder therapies. Although cinnamon has been reported in various studies as a promotor of metabolic health, there is contrasting evidence from trials. Gou et al. performed a systematic review to evaluate the impact of cinnamon supplementation on metabolic outcomes in patients with metabolic diseases. The authors found an association of cinnamon consumption with improved fasting glucose and lipid profiles. Interestingly, the effects were higher in patients with diabetes and metabolic syndrome. Moreover, subgroup analysis suggests that doses >1.5 g/day in interventions of short duration (≤2 months) may enhance these benefits. The review paper produced by Lin et al. showed that resistant starch (RS) consumption (around 30 g/day and lasting over 8 weeks) significantly reduces hip circumference, total cholesterol, low-density lipoprotein cholesterol, and improves superoxide dismutase levels. However, the effects of RS on waist circumference, fasting insulin, HOMA-IR, and TNF-α were less conclusive. Ten-weeks treatment with an extract of Pangxiejiao plant, which is rich in flavonoids, polysaccharides, triterpenoids, etc., protected from BW gain, fasting blood glucose, glucose intolerance, and dyslipidemia induced by high-fat and high-fructose diet consumption. Along with these changes, decreased serum levels of inflammatory markers like interleukin (IL)-1β, IL-6, and tumor necrosis factor (TNF)-α were observed. Furthermore, these benefits were associated with a decrease of the *Firmicutes*/*Bacteroidetes* ratio, suggesting an involvement of the gut microbiota in the metabolic benefits of Pangxiejiao (Wang W. et al.).

Metabolic disorders are highly correlated with liver impairment, which is typically caused by modern dietary practices. Six papers discussed the use of functional components to alleviate liver injury. The study conducted by Xu et al. showed that Alternative Healthy Eating Index (AHEI), Healthy Eating Index-2020 (HEI-2020), Dietary Approaches to Stop Hypertension Index (DASHI), and Mediterranean Diet Index (MEDI) were found to be negatively associated with metabolic dysfunction-associated steatotic liver disease (MASLD) risk. All AHEI, HEI-2020, DASHI, and MEDI encourage eating more vegetables, fruits, whole grains, nuts, proteins, and mono- and polyunsaturated fatty acids while restricting the consumption of unhealthy foods. In this line, Zheng et al. found a negative association between Vitamins A, C, E, and carotenoids with fatty liver index (FLI) in females, but only vitamins A and E were negatively associated in males, indicating sex-specific effects. Park and Peters conducted a study on the *in vivo* effects of the bioactive compounds Javamide-I/-II found in coffee, which have been proven to have anti-inflammatory properties. Rats that were treated for 16 weeks showed hepatic protection and a reduction in inflammatory markers. The absence of toxicity means that Javamide-I/-II can be used as a treatment for metabolic disorders. In another study, 3 weeks of pretreatment with N-acetylcysteine, a precursor of L-cysteine and glutathione, protected from hepatic and muscle LPS-induced inflammation in piglets as shown by reduced inflammatory makers including IL-1β, interferon-γ (IFN-γ), and H_2_O_2_ (Li P. et al.). Hakkak et al. evaluated the effect of dietary soy protein on steatosis and microbiota profile in obese Zucker rats. They found that the treatment reduces steatosis and enriches *Muribaculaceae* groups, composed of short-chain fatty acids-producer microbes, suggesting that dietary soy protein decreases steatosis by modulating gut microbiota. Finally, Wang Yif. et al. tested the protective effect of the food formula GGV (glutathione, Ganoderma lucidum extract, and vitamin C). Mice previously treated with CCl4 (carbon tetrachloride) were given GGV and evaluated for liver markers of dysfunction and changes in gut microbiota. GGV treatment significantly decreased CCl4-induced steatosis and oxidative stress, as well as increased the abundance of the health-promoting Akkermansia. The potential of this food formula in combating hepatic dysfunction is demonstrated by these results.

T2D is characterized by altered metabolic control. Ketogenic diets (KD) replace glucose with ketone bodies, becoming an interesting nutritional strategy for managing diabetes. Li Z. et al. conducted a systematic review of the current research status and hotspots of KD in diabetes management. From 2005 to 2024, the United States was the top country in terms of the number of published papers, followed by China, Australia, and Canada. The focus of current research is on the impact of KD on blood glucose control, insulin resistance, and lipid metabolism in diabetic patients. Mechanistic studies on KD in diabetes management concentrate on aspects such as gene regulation by hydroxybutyrate, anti-inflammatory effects, and oxidative stress. The gut microbiome is becoming a significant research area. In another study, Michel et al. evaluated the anti-hyperglycemic effect of Amla and olive fruit extracts in hyperlipidemic adults with prediabetes and T2D. The treatment showed a reduction in blood glucose of 27.9 and 4.7% in pre-diabetic and diabetic groups, respectively.

The functional effects of various prebiotics in the context of cardiovascular diseases have been discussed in five articles. Hossain et al. compared the oat polar lipids and sunflower lecithin post-prandial effects on cardiometabolic parameters in healthy young adults. Compared to rapeseed oil (control group), both oat polar lipids and sunflower lecithin improved post-prandial glucose and insulin. The presence of decreased triglycerides and increased GLP-1 levels was observed as well. The recommendation of consuming polar lipid sources is highlighted by these results. Puerarin, an isoflavone derivative used in traditional Chinese medicine, enhances lipid metabolism and decreases lipid absorption. The review of literature by Ou et al. revealed that puerarin has cardioprotective benefits across multiple trials. Due to its low bioavailability, most studies showing such benefits administered the compound intravenously. Despite being beneficial for metabolism, puerarin treatment had side effects, which included drug fever, rash, nausea, vomiting, diarrhea, hepatic/renal damage, palpitations, anaphylactic shock, and hemolysis. The challenge now is to find a safer delivery system that allows us to profit from its benefits. The antihypertensive effects of garlic have been well-demonstrated. However, there is substantial heterogeneity in the current literature. To overcome this issue, Tang et al. performed a specific assessment of the antihypertensive effects of garlic treatment by focusing specifically on hypertensive patients. The authors systematically reviewed 10 articles fitting the inclusion criteria. Globally, data showed reduced blood pressure and Tnf-α levels along with increased levels of high-density lipoproteins. However, potential gastrointestinal adverse effects should be considered in clinical practice. Wang X. et al. carried out a comprehensive review to discuss the potential neuroprotective effect of the natural carotenoid astaxanthin in ischemic stroke, focusing on its mechanisms involving antioxidation, anti-inflammation, enhancement of DNA repair, protection of blood–brain barrier, and promotion of neuronal survival. The authors conclude that astaxanthin has the potential to be used as therapy for ischemic stroke, however, detailed studies are required to establish safe doses and treatment conditions. Finally, Wang G. et al. sought to investigate the effect of single vs. joint dietary vitamins on the prevalence of cardiovascular disease in chronic kidney disease patients through a cross-sectional analysis. They observed that high intake of vitamin B6 and vitamin E was associated with reduced cardiovascular risk. Interestingly, co-exposure to nine dietary vitamins (vitamins A, B1, B2, B6, B12, C, D, E, and K) was negatively correlated with cardiovascular risk.

Hyperuricemia is a highly prevalent metabolic disorder characterized by high serum uric acid levels, which result from dysregulated purine metabolism or impaired uric acid excretion. This medical condition is a risk factor for other metabolic diseases like cardiovascular disease and T2D. In this topic, three papers investigated functional solutions for hyperuricemia. Huang et al. generated a mini review of the literature aiming at understanding the traditional Chinese medicine (TCM) potential as an alternative to pharmacological therapies which are associated with various side effects. The review highlights TCM as an effective approach, sometimes with better effects than Western drugs. However, the study highlights that further preclinical and clinical studies are required to establish effective doses, individualized interventions, toxicity, etc. In the same line, Wang Yix. et al. in a cross-sectional study investigated the relationship between probiotics and prebiotics intake and the prevalence of hyperuricemia. Probiotic consumption was associated with lower hyperuricemia. In contrast, prebiotics did not show an effect on blood uric acid. Prebiotic was mainly based on saccharides; this could explain the contrasting result with the previous review paper. In line with the latter study, Zhang et al. demonstrated that *Lactobacillus paracasei* N1115 reduced serum uric acid, protected renal function, and attenuated inflammation. The mechanism involved in these effects includes the regulation of xanthine oxidase and reshaping the gut microbiota, with increased abundance of *Bifidobacterium*. These findings indicate that prebiotic and probiotic-based treatments can be used to manage hyperuricemia.

In addition to the articles discussed above, four articles investigated medical conditions associated with metabolic dysfunction, such as diarrhea (Wu et al.), osteopenia/osteoporosis (Li D. et al.), Parkinson's disease (Jia H. et al.), and lipid metabolism in mammary gland epithelial cells (Che et al.).

In summary, the collection of original and review articles covering this Research Topic supply invaluable data that provides further biological basis for the use of functional foods for metabolic health. According to the research, both probiotics and prebiotics promote beneficial metabolic changes in different tissues, which are significantly mediated by the gut microbiota. Both protection and corrective effects were achieved by functional foods or functional compounds. This suggests that these types of foods should be consumed by both healthy people and those with metabolic disorders. Although the beneficial effects of functional foods were demonstrated by a considerable number of studies presented in this Research Topic, further research is needed to address relevant aspects such as safe dosages, duration of intervention, and routes of administration. Based on the article included in this topic by Shi et al., which shows that research on nutritional supplements is in full development, it is expected that these aspects will be intensively investigated shortly and that therapies based on functional foods will be consolidated.
